# Integrating a brief alcohol intervention with tobacco addiction treatment in primary care: qualitative study of health care practitioner perceptions

**DOI:** 10.1186/s13722-021-00225-x

**Published:** 2021-03-16

**Authors:** Nadia Minian, Aliya Noormohamed, Mathangee Lingam, Laurie Zawertailo, Bernard Le Foll, Jürgen Rehm, Norman Giesbrecht, Andriy V. Samokhvalov, Dolly Baliunas, Peter Selby

**Affiliations:** 1 Nicotine Dependence Service, Centre for Addiction and Mental Health, 1025 Queen Street W, Toronto, ON M6J 1H4 Canada; 2 Department of Family and Community Medicine, University of Toronto, 500 University Avenue, Toronto, ON M5G 1V7 Canada; 3 Campbell Family Mental Health Research Institute, Centre for Addiction and Mental Health, 250 College St, 1st floor Toronto, ON M6J 1H4 Canada; 4 Institute of Medical Science, Faculty of Medicine, University of Toronto, 1 King’s College Circle, Toronto, ON M5S 1A8 Canada; 5 Department of Pharmacology and Toxicology, Faculty of Medicine, Medical Sciences Building, University of Toronto, Room 4207, 1 King’s College Circle, Toronto, ON M5S 1A8 Canada; 6 Department of Psychiatry, University of Toronto, 250 College St., Toronto, ON M5T 1R8 Canada; 7 Institute for Mental Health Policy Research, Centre for Addiction and Mental Health, 33 Russell Street, Toronto, ON M5S 2S1 Canada; 8 Technische Universität Dresden, Klinische Psychologie & Psychotherapie, Chemnitzer Str. 46B, 01187 Dresden, Germany; 9 Dalla Lana School of Public Health, University of Toronto, 155 College Street, Toronto, ON M5T 3M7 Canada; 10 Addiction Division, Centre for Addiction and Mental Health, 33 Russell Street, Toronto, ON M5S 2S1 Canada; 11 Homewood Health Centre, 150 Delhi St., Guelph, ON N1E 6K9 Canada; 12 Department of Psychiatry and Behavioural Neurosciences, McMaster University, 100 West 5th Street, Hamilton, ON L8N 3K7 Canada; 13 School of Public Health, The University of Queensland, Herston, QLD Australia

**Keywords:** Alcohol drinking, Tobacco, Smoking cessation, Primary Health Care, Qualitative Interviews, Clinical Decision Support System, Hexagon Tool

## Abstract

**Background:**

Randomized trials of complex interventions are increasingly including qualitative components to further understand factors that contribute to their success. In this paper, we explore the experiences of health care practitioners in a province wide smoking cessation program (the Smoking Treatment for Ontario Patients program) who participated in the COMBAT trial. This trial examined if the addition of an electronic prompt embedded in a Clinical Decision Support System (CDSS)—designed to prompt practitioners to Screen, provide a Brief intervention and Referral to Treatment (SBIRT) to patients who drank alcohol above the amounts recommended by the Canadian Cancer Society guidelines—influenced the proportion of practitioners delivering a brief intervention to their eligible patients. We wanted to understand the factors influencing implementation and acceptability of delivering a brief alcohol intervention for treatment-seeking smokers for health care providers who had access to the CDSS (intervention arm) and those who did not (control arm).

**Methods:**

Twenty-three health care practitioners were selected for a qualitative interview using stratified purposeful sampling (12 from the control arm and 11 from the intervention arm). Interviews were 45 to 90 min in length and conducted by phone using an interview guide that was informed by the National Implementation Research Network’s Hexagon tool. Interview recordings were transcribed and coded iteratively between three researchers to achieve consensus on emerging themes. The preliminary coding structure was developed using the National Implementation Research Network’s Hexagon Tool framework and data was analyzed using the framework analysis approach.

**Results:**

Seventy eight percent (18/23) of the health care practitioners interviewed recognized the need to simultaneously address alcohol and tobacco use. Seventy four percent (17/23), were knowledgeable about the evidence of health risks associated with dual alcohol and tobacco use but 57% (13/23) expressed concerns with using the Canadian Cancer Society guidelines to screen for alcohol use. Practitioners acknowledged the value of adding a validated screening tool to the STOP program’s baseline questionnaire (19/23); however, following through with a brief intervention and referral to treatment proved challenging due to lack of training, limited time, and fear of stigmatizing patients. Practitioners in the intervention arm (5/11; 45%) might not follow the recommendations from CDSS if these recommendations are not perceived as beneficial to the patients.

**Conclusions:**

The results of the study show that practitioners’ beliefs were reflective of the current social norms around alcohol use and this influenced their decision to offer a brief alcohol intervention. Future interventions need to emphasize both organizational and sociocultural factors as part of the design. The results of this study point to the need to change social norms regarding alcohol in order to effectively implement interventions that target both alcohol and tobacco use in primary care clinics.

*Trial registration* ClinicalTrials.gov NCT03108144. Retrospectively registered 11 April 2017, https://www.clinicaltrials.gov/ct2/show/NCT03108144

## Background

Tobacco use and alcohol consumption are among the leading causes of chronic disease-related morbidity and mortality [1, 2]. Both substances have been linked to a wide range of chronic disease-related harms and have significant social, economic, and health impacts [3, 4]. Moreover, the combination of tobacco and alcohol use has a multiplicative risk of aero-digestive cancers [5–12]. This is especially concerning as smokers are more likely to consume alcohol compared to non-smokers [13].

Screening, Brief Intervention, and Referral to Treatment (SBIRT) is a program to reduce unhealthy alcohol use, based on the evidence-based practice of Screening and Brief Intervention [14–19]. Despite SBIRT’s known efficacy, the majority of healthcare practitioners in Ontario do not incorporate brief alcohol interventions into their practice [20]. This gap is especially relevant in smoking cessation treatment among individuals who also consume alcohol; given the association between the two substances [13, 21]. Moreover, drinking alcohol is a barrier to successful smoking cessation and vice versa [22–26]. Therefore, an integrated approach to treatment would be ideal when providing care to dual tobacco and alcohol users.

With funding from the Canadian Cancer Society Research Institute, a cluster randomized trial named “Personalized patient alerts and care pathways to prompt prevention interventions for combined alcohol and tobacco users in primary care (COMBAT)” was designed (ClinicalTrials.gov # NCT03108144) [27]. The primary aim of the trial was to assess whether a web-based clinical decision support system (CDSS) guiding practitioners to conduct SBIRT influenced the proportion of practitioners delivering a brief alcohol intervention to their patients who were smoking and drinking above the Canadian Cancer Society (CCS) guidelines; female ≥ 1 standard (13.6 g) drink/day; men: ≥ 2 standard drinks/day (operationally defined for this study as: women consuming seven or more, and men consuming fourteen or more alcoholic beverages in the past week) [27, 28].

A CDSS is an information system designed to present patient-specific, actionable information to help health care practitioners make diagnosis and treatment decisions [29, 30]. Within our design, the CDSS supports the delivery of SBIRT by automating screening for at-risk drinking, offering recommendations for the type of brief intervention to deliver, and suggesting resources to refer the patient for further treatment. The CDSS was implemented in the Smoking Treatment for Ontario Patients (STOP) program as a part of their online portal (STOP Portal), which is used by all health care practitioners implementing the STOP program (referred to as STOP practitioners) to enroll patients and record the treatment provided. The STOP program provides patients with behavioural counselling for smoking cessation and up to 26 weeks of nicotine replacement therapy over the course of the program (12 months). Treatment is tailored by STOP practitioners, who will usually meet with their patients every 2–4 weeks. STOP practitioners may dispense up to 4 weeks of NRT at any given visit with the patient.

For the COMBAT Trial, a total of 221 primary care clinics participating in the STOP program were randomized to the intervention arm or control arm. STOP practitioners in clinics assigned to the intervention arm had automated screening for alcohol use and received a prompt, in the STOP Portal, when a patient reported consuming alcohol above CCS guidelines. STOP practitioners in clinics assigned to the control arm did not receive a prompt when a patient reported consuming alcohol above CCS guidelines; however the same screening questions and resources for alcohol use were available to them. STOP practitioners come from a wide variety of disciplines; including registered nurses, doctors, nurse practitioners and social workers. For the COMBAT Trial, STOP practitioners were asked to screen their STOP patients for alcohol use and provide a brief intervention to those who were drinking above CCS guidelines. STOP patients from clinics participating in the COMBAT Trial, who drank above CCS guidelines, were part of the trial.

The implementation of the COMBAT Trial was guided by the Interactive Systems Framework (ISF) for Dissemination and Implementation [31], which states that three systems are needed to implement scientific knowledge: the support system, the synthesis and translation system, and the delivery system. As a part of the support system, and before the launch of the trial, all STOP practitioners were invited to participate in two 1-h-long interactive web-based SBIRT trainings. The webinars can be accessed at: https://tinyurl.com/555z2nbq and https://tinyurl.com/2wt8abrs. For the synthesis and translation system, we created and distributed knowledge translation products (e.g. infographics, newsletters, and a slide deck) to practitioners. These knowledge translation products contained latest available evidence on the risks of concurrent alcohol and tobacco use. The primary care clinics implementing the STOP program were the ISF delivery system.

The results of the trial showed that 99.6% of patients were screened for alcohol use. Moreover, a brief alcohol intervention and an educational resource for alcohol use was offered to 45% of patients who reported drinking above CCS guidelines [32]. The results also showed that the use of a prompted CDSS:

1. Did not increase practitioner likelihood of offering an alcohol intervention to eligible patients [32].
2. Increased the acceptance rate of an educational alcohol resource by patients offered a resource by their practitioner. If practitioners had access to the prompted CDSS, their patients were significantly more likely to accept an educational resource [32], and these results were not moderated by sex of the patient [33].
3. Did not influence patients’ smoking status and alcohol consumption within CCS guidelines at 6-month follow-up [32].

There is growing acknowledgement that qualitative methods can help with understanding why complex interventions, such as those employed in implementation science, are successful or unsuccessful [34–36]. The perspectives of practitioners can provide the necessary context for the results observed in the trial. As a result, qualitative interviews were conducted with STOP practitioners to understand their experience with the trial, including the facilitators and barriers to delivering alcohol interventions in a smoking cessation program, and the practitioners’ perceptions on the role of the CDSS. STOP practitioners from both the intervention and control arms of the COMBAT trial were interviewed to assess any similarities or differences in the barriers and facilitators they experienced. The aim of the current study was to examine the factors influencing implementation and acceptability of the COMBAT trial among STOP practitioners. The results of this study will help inform the development of interventions aimed at incorporating alcohol screening and brief intervention into a smoking cessation program delivered in primary care settings.

## Methods

### Participants

Participants of this qualitative study were 23 STOP practitioners that were part of the COMBAT Trial who had enrolled at least 10 patients drinking above CCS guidelines into the STOP program during the trial period. Participants were selected using stratified purposeful sampling [37] to capture meaningful variation between the following categories, relevant to the study’s research objectives:

- Practitioners who provided a resource to appropriate patients most of the time (at least 70%), some of the time (30%-69%), or infrequently (less than 30%).
- Each study arm: practitioners who worked in clinics that were randomized to the intervention or control arm of the COMBAT trial.
- Each organization type: Family Health Team (FHT), Community Health Centre (CHC) and Nurse Practitioner-Led Clinic (NPLC).

Stratified purposeful sampling involves sampling individuals that are especially knowledgeable about or have experienced with the topic of interest [38], in this case, we wanted to ensure that we interviewed HCPs with the different experiences identified above.

Twenty-five potential participants received a phone call describing the project and inviting them to participate in an interview. This qualitative study, including the recruitment and consent process, received ethics approval from the Centre for Addiction and Mental Health Research Ethics Board (#035–2015).

### Data collection

All interviews were conducted between February and April 2017 by ML via telephone. Each interview lasted between 45 and 90 min. The interviews explored practitioners’ experiences with delivering an alcohol intervention as part of the STOP program, including facilitators and barriers (Additional File 1: Interview Guide). The interview questions were structured around the six components of the original National Implementation Research Network’s Hexagon Tool: evidence, resource availability, readiness, needs, fit, and capacity to implement [39, 40]. While this tool is primarily designed to guide decisions on the type of evidence-based intervention to implement, it can also be used post-implementation to evaluate an intervention [41, 42]. Interviews were audio recorded and transcribed verbatim by a transcriptionist. These transcripts were cross‐checked with interview audio files and verified for accuracy by a researcher. Audio files and transcripts were anonymized and stored in a secure online database.

### Data analysis

Data was coded and analyzed using the framework analysis approach [43]. Framework analysis is a qualitative method well suited for studies that use a structured topic guide that aims to identify patterns within the data and has been commonly used in health care research studies [43]. Transcripts were imported into NVivo 11 [44] and a preliminary coding structure was developed using the updated Hexagon Tool framework released in 2018 [45], as well as key concepts from the interview guide. A subset of the transcripts was coded separately by two researchers in NVivo; additional codes were then added and revised iteratively as new themes arose during analysis. Any emergent themes were discussed and coding discrepancies were resolved through discussion until consensus was reached, followed by further revision of the coding framework. Inter rater reliability was checked using NVIVO’s comparison query, which calculates percentage agreement and the kappa coefficient [44]. This process was repeated for three rounds until inter-rater agreement of at least 90% was achieved and we had a kappa value of at least 0.76. At this point, the coding structure was finalized and no additional codes were added. All interviews were re-coded using the final framework. Data was organized into a Framework Matrix in NVivo; individual cases (practitioners) were sorted by row, while themes were placed in the columns of the matrix, with each intersecting cell summarizing the content for that practitioner and theme. Themes specific to the study arm were also analyzed by creating a separate matrix for each study arm. In these matrices, practitioners were also sorted by row and themes were placed in the columns of the matrix. The intersecting cell summarized the content for that practitioner and theme, and the matrix as a whole only had the content of one study arm.

Throughout the analysis and writing of this manuscript, the research team was reflective of their status (e.g. working for the organization that coordinates the STOP program), their training (e.g. implementation science and the Hexagon Tool), and their expertise in tobacco and alcohol control.

## Results

Of the 25 providers invited to participate in this study, 23 agreed to participate and completed an interview, yielding a response rate of 92%. Participants’ baseline characteristics are shown in Table 1.

**Table 1:** Baseline characteristics of the 23 practitioners who participated in the interviews

	Control (n = 12) n (%)	Intervention (n = 11) n (%)
Organization type		
Community Health Centre	2 (17%)	5 (45%)
Family Health Team	9 (75%)	5 (45%)
Nurse Practitioner Led Clinic	1 (8%)	1 (9%)
Organization performance		
Clinics that provided the alcohol resource infrequently (less than 30%)	4 (33%)	3 (27%)
Clinics that provided the alcohol resource some of the time (30%-69%)	7 (58%)	6 (55%)
Clinics that provided the alcohol resource most of the time (at least 70%)	1 (8%)	2 (18%)
Practitioner gender		
Male	1 (8%)	1 (9%)
Female	11 (92%)	10 (91%)
Practitioner occupation		
Nurse^a^	6 (50%)	7 (64%)
Pharmacist	2 (17%)	2 (18%)
Other^b^	4 (33%)	2 (18%)
Years practitioner has been involved in Smoking Treatment for Ontario Patients program		
< 2 years	2 (17%)	2 (18%)
2–5 years	3 (25%)	6 (55%)
> 5 years	7 (58%)	3 (27%)

^a^Includes registered nurse and registered practical nurse

^b^Includes social worker, respiratory therapist, dietician

**Figure 1.**
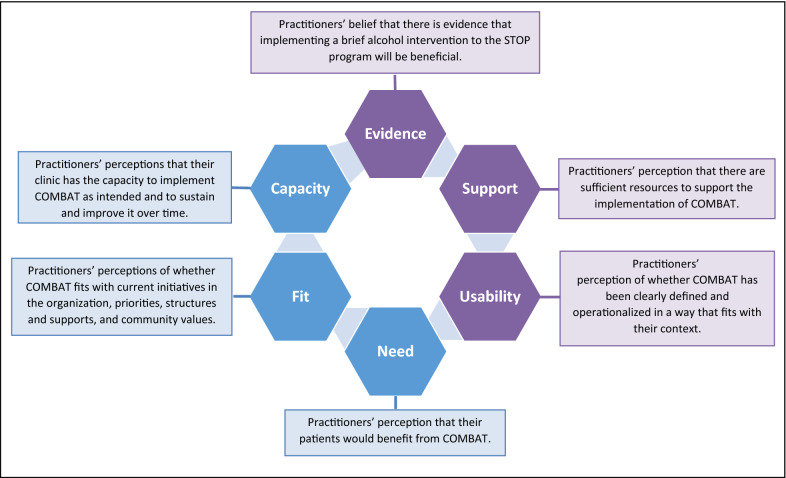
Domains used to organize the emerging themes. Adapted from National Implementation Research Network’s Hexagon tool

We organized our findings below using the key domains of the Hexagon Tool [45]. First, we present the three components associated with the COMBAT program (evidence, supports, and usability) followed by the three components associated with the STOP-implementing clinics (need, fit, capacity). Please see Fig. 1 for a visual depiction of these domains.

(1) Evidence: defined as practitioners’ belief that there is evidence that implementing a brief alcohol intervention to the STOP program will be beneficial.

The CDSS did not seem to play any role in participants’ belief that there is evidence that implementing a brief alcohol intervention to the STOP program will be beneficial. Most practitioners from both arms (n = 17, 74%) reported they were aware of, and agreed with, the evidence demonstrating the effectiveness of addressing alcohol in a smoking cessation program. However, several practitioners (n = 13, 56%) expressed some concerns with using the CCS guidelines to screen for alcohol use:

"I think that, you know, guidelines aren’t black and white. Right?… I think there needs to be some professional judgment." Interview 21

A few practitioners from both arm (n = 6, 26%) voiced concerns that addressing alcohol at the same time as smoking may negatively affect patients’ smoking cessation efforts.

“If you [try to] fix everything at once they end up not doing anything. They get discouraged and they get dismayed and they just, fall off wagon… the alcohol thing, I really don't think it's something to be pursued at …that baseline appointment. Yeah. Mention it but other than that, move on and let's stick with your priority because, you've already identified the...that you want to give up smoking.” Interview 10

(2) Supports: defined as practitioners’ perception that there are sufficient resources to support the implementation of COMBAT, including staff, training, technology supports, data systems and administration.

Most practitioners from both arms mentioned that even though there were some resources in place at their clinics to support the implementation of COMBAT, much more was needed. Organizational limitations were frequently cited as a barrier to providing a brief alcohol intervention. Many practitioners (n=18, 78%) pointed to time as the most prominent barrier for implementing the COMBAT intervention.

“I feel the questions are relevant, the intervention is relevant. It’s just a matter of whether or not we’re able to do it in that given timeframe.” Interview 20

In order to address the time barrier, some practitioners in both arms (n=7, 30%) reported that their organization implemented an adaptation to address time constraints, including lengthening the initial appointment, allocating extra time each day for completing documentation and changing scheduling practices. The majority of these seven practitioners (n=5, 71%) were from organizations randomized to the control arm of the study.

Training was another issue that was frequently reported as a major barrier for implementing COMBAT. Most practitioners interviewed (n=19, 83%) expressed desire for more training; however, the majority of these 19 practitioners (n=15, 79%) had not attended any of the online training webinars offered by the study team. Practitioners who viewed the webinar had more favourable attitudes towards implementing the COMBAT initiative, and some credited this directly to watching the webinar:

“The webinar I listened to help reinforce that it’s not a separate issue. They can address both at the same time and people can be successful at addressing both two addictions." Interview 18

Many respondents in both arms (n=19, 83%), including those who attended the webinar, expressed a need for further training on techniques and tools that can be used when addressing alcohol and tobacco.

“It would be nice to have maybe even like a lunch webinar or something like that, just to kind of provide some more education.” Interview 22

Practitioners in the intervention arm had mixed views about the CDSS. Most practitioners in the intervention arm (n=9, 82%) reported they found the prompted CDSS guidance helpful, and that it helped them deliver brief interventions when needed.

“I think as far as the portal goes, it’s easy-peasy. I mean it’s very easy read, it’s very easy fill in the blanks, and I mean to be me it’s got all the options … the reminders to give an [alcohol] intervention are great” Interview 11

However, some practitioners in the intervention arm (n=6, 55%) expressed some challenges with using the portal, including: questioning the accuracy of the CDSS scoring, experiencing portal slowdowns, or experiencing discomfort when a patient was able to view the practitioner’s screen with CDSS messaging.

"It could be my calculations are off, but I just wondered sometimes if it triggered some of them for being over when they weren't." Interview 17

“Sometimes I’m like, ‘Oh, you know, are the patients seeing this.’ Are they, you know, now going to be on guard that they see this popup that it’s indicating that they have an alcohol addiction problem.” Interview 15

A few (n=3, 27%) of practitioners in the intervention arm mentioned that they would ignore the prompts especially when they doubted the accuracy of the CDSS recommendation, when they did not feel that they had enough training or felt they did not have time to address alcohol properly, or when they felt that the patients are not receptive to receiving an alcohol intervention:

“It's asking me to perform a brief intervention … and I'm not going to do that because I'm not trained.” Interview 10

“I have to say it (deciding whether to provide a brief alcohol intervention when prompted by the CDSS) comes down to time. … I have this, my days are booked solid, and so, you know, to go over 10 minutes with somebody puts me behind. Like it just compounds my day, right? So I always have to be conscious of my time, and so I sometimes just don’t have the time.” Interview 8

(3) Usability: defined as practitioners’ perception of whether COMBAT has been clearly defined and operationalized in a way that fits with their context.

There were mixed reviews regarding the operationalization of the COMBAT initiative and its usability. Eighty-three percent (n=19) of practitioners found screening for alcohol was useful and they were happy to use validated assessments that could allow patients to reflect on their drinking.

“I think it’s been really good, because there is more of an awareness on the client’s perspective … the questions are a whole lot more specific.” Interview 3

“It’s nice to be able to point out some facts about the risks of cancer and alcohol use, and offer support around that. Because people won’t realize if they’re drinking too much until it comes up.” Interview 16

A common concern for practitioners working in clinics randomized to the intervention arm (n=7, 64%) was that the CCS guidelines adopted by COMBAT were restrictive, and did not reflect social norms.

“It [the STOP Portal] warns you that they're drinking over the limit … And, they don't see that. And, I think society maybe we don't see that as a problem” Interview 10

“Patient’s may only drink once a week but on those occasions they might have five or more drinks and this triggers a whole line of questioning that I’m not sure is completely appropriate.” Interview 12

Some practitioners in the intervention arm (n=9, 39%) worried that if they intervene as prompted, it might end up stigmatizing patients.

“I just worry that if they're going to have to worry about me ask them about their drinking which is the way some people perceive it, then they're not going to come back to me for smoking. So I don't want to set that stigma.” Interview 17

Despite being offered training prior to the initiative launch, most practitioners in the control arm did not seem aware of the CCS guidelines. When asked how they identified patients drinking above guidelines, many practitioners in the control arm (n=9, 75%) reported using informal ways to score patients drinking.

“I don’t add it up … And I don’t report a specific number … in my EMR encounter. I just mention alcohol use.” Interview 13

"I base it on the answers that they’ve given me throughout these questions. And you know, I’ve asked them if they feel alcohol is an issue." Interview 6

The few practitioners (n=3, 25%) in the control arm who reported using a scoring system used Canada’s Low-Risk Drinking Guidelines.

“So, what I use are the, what’s it called, the ‘rethink your drinking’ or the low risk drinking guidelines. That is what I use. So, if people are above that I tell them they are having too many drinks at a time.” Interview 19

(4) Needs: defined as practitioners’ perception that their patients would benefit from COMBAT.

Practitioners were asked about the need for an alcohol intervention among STOP program patients. Most participants in both arms (n=18; 78%) felt an alcohol intervention was needed due to: its connection with smoking behaviours; prevalence of high drinking levels in their patient populations; and the frequent underestimation of drinking risks in primary care. Only one practitioner mentioned there is a need for this intervention in order to reduce cancer risks.

"I think it’s very relevant. I think it’s an important conversation to have, particularly in primary care just in general." Interview 23

“How important it truly is in its relation specifically to smoking cessation … The two go hand-in-hand ... And, often the biggest reason why people, especially young people, don’t quit is because of alcohol.” Interview 4

A few practitioners from both arms (n=5, 22%) felt there was no need to address alcohol in the STOP program since their patients were facing more pressing issues, and that asking questions about alcohol consumption could cause unexpected troubles and shift the focus away from smoking cessation:

“There are more important issues right now than alcohol. It’s not that much of a big issue in our centre We do have an increase in diabetes in our community and COPD. So, alcoholism is not a big issue. So, these questions have you know, they’re there. I need to do them but they’re not really helping in any way." Interview 7

“It opens another Pandora box I guess, right?” Interview 8

(5) Fit: defined as practitioners’ perceptions of whether COMBAT fits with current initiatives in the organization, priorities, structures and supports, and community values.

All participants commented that the COMBAT initiative fits well with the priorities of their clinic and with the STOP program.

"I feel that the smoking cessation encompasses their alcohol, their eating, their sleeping, their…everything’s a part of it, so I feel that I’m doing diligence if I offer them, you know, more information about some of the things that might arise because of their smoking cessation or something that’s already a co-addiction" Interview 2

“When you get into smoking cessation, of course we all know the relevance [of addressing alcohol]” Interview 14

However, some practitioners from both arms were concerned that even though the alcohol intervention fit well with their clinics, it did not fit well with some of their patients. Respondents from both the intervention and control arm (n=11, 48%) reported that addressing alcohol with their clients was very difficult because alcohol is so normalized in our society.

"The men, obviously more than the women, are the heavy smokers of two packs a day. You know, drink a 24 on the weekend kind of thing. Or come home from hunting and drink three or four beer every night kind of thing. So, it’s certainly an excess and they know that but they’re not going to...that’s what they’ve done, that’s what their father did, that’s what their son did; that’s just what they do." Interview 17

Some practitioners (n=7, 30%) also described social determinants of health as barriers to the appropriateness or fit of this intervention among their patient populations.

“We do have a lot of people on social assistance, low education, low income who have a lot of other social determinants of health issues that they’re struggling with, and I feel like that’s definitely a barrier… their lives are too overwhelming to take it on.” Interview 9

The CDSS did not seem to play a role on practitioner’s perceptions of whether COMBAT fits with current initiatives in their organization.

(6) Capacity to implement: defined as practitioners’ perceptions that their clinic has the capacity to implement COMBAT as intended and to sustain and improve it over time.

Lack of trained staff as well as lack of supports in the geographical area were mentioned as a common barriers for implementing the COMBAT initiative:

"We only have one Social Worker; … wait time for people in our area to get mental health counseling can be up to six months…we’ve ripped off the Band-Aid and we have no way of stopping the bleeding at this point." Interview 12

A common phrase that came up when practitioners explained they lacked the capacity to implement COMBAT with fidelity, especially to provide the brief intervention, was they felt that they were ‘opening up a can of worms’.

“Now I've opened a whole can of worms, now what? They came to see me for smoking and now I know about drinking...it's a lot.” Interview 5

"So, if you identify it, then you’ve opened up a full can of worms and then you need to do something with it...because now you own it … It’s great to identify the issues but what’s going to be in place to deal with it?" Interview 12

“It's like opening up another can of worms. And, right now we're trying to deal with the smoking cessation, right? Like, one thing at a time.” Interview 9

Lack of trained staff as well as lack of supports in the geographical area were mentioned as a common barriers for implementing the COMBAT initiative:

"We only have one Social Worker; … wait time for people in our area to get mental health counseling can be up to six months…we’ve ripped off the Band-Aid and we have no way of stopping the bleeding at this point." Interview 12

A common phrase that came up when practitioners explained they lacked the capacity to implement COMBAT with fidelity, especially to provide the brief intervention, was they felt that they were ‘opening up a can of worms’.

“Now I've opened a whole can of worms, now what? They came to see me for smoking and now I know about drinking...it's a lot.” Interview 5

"So, if you identify it, then you’ve opened up a full can of worms and then you need to do something with it...because now you own it … It’s great to identify the issues but what’s going to be in place to deal with it?" Interview 12

“It's like opening up another can of worms. And, right now we're trying to deal with the smoking cessation, right? Like, one thing at a time.” Interview 9

## Discussion

These interviews provide a better understanding of issues that should be considered when implementing an SBIRT-based intervention to address risky alcohol use within the context of a smoking cessation treatment setting. In our study, practitioners generally reported positive attitudes toward the inclusion of alcohol screening questions, and reported systematically using these questions to screen their patients for risky alcohol use. Most practitioners felt addressing alcohol in smoking cessation treatment was important and effective. However, many practitioners were not always in agreement with, or aware of, the established alcohol guidelines, and reported using subjective judgement or non-systematic methods of assessing whether or not to provide a brief intervention alcohol intervention. When they did report using a guideline to score patients’ answers, it was the Low-Risk Drinking Guidelines [46] which have higher drinking cut-offs than the CCS drinking guidelines. Similar to other studies, our study showed that practitioners’ decisions to provide a brief intervention are heavily influenced by the social acceptability of alcohol, which acts as a barrier to practitioners offering brief interventions [47–49].

Moreover, the majority of practitioners in our study identified lack of training as a major barrier in addressing alcohol as a part of smoking cessation; which is similar to what has been reported in other studies [49–51]. While only a few practitioners had attended at least one of the two webinar trainings provided by COMBAT, practitioners who had attended a webinar still expressed a need for more training. Unlike what has been observed in the literature [52], online webinar trainings did not appear sufficient in helping practitioners feel prepared to deliver SBIRT. Given that lack of training was identified as a major barrier, it may be important for future programs to invest additional resources for more intensive forms of trainings (i.e. longer duration, in-person) to increase practitioners’ confidence and capacity to deliver the intervention.

Also comparable to what has been observed in other studies, practitioners’ fears of compounding multiple risk factors into one intervention strongly influenced their decision to provide an alcohol intervention as part of smoking cessation treatment. Practitioners acknowledge their patients have multiple comorbidities and socioeconomic barriers to successfully quitting smoking, and fear that adding behavioural interventions to smoking cessation programming could overwhelm or discourage patients from making any positive change. Anticipating hesitation from practitioners, COMBAT trainings communicated that best practice guidelines [53, 54] recommend concurrent treatment of tobacco and risky alcohol use. Future trainings and brief intervention scripting need to include more effective messaging about the importance and benefit of addressing alcohol and smoking simultaneously to alleviate practitioner (and perceived patient) concerns.

In contrast to previous studies [49, 55], practitioners reported the screening questions were useful for understanding their patients’ alcohol consumption. Moreover, practitioners participating in the COMBAT trial did not engage mechanically with the CDSS. As has been reported elsewhere [56], practitioners would only follow the CDSS instruction if they believed it to be accurate and an appropriate treatment approach for their patients.

These findings help us understand some of the quantitative findings of the trial, including the observation that most patients were screened for alcohol use (99.6%) but fewer were offered a brief intervention [32]. The results of this qualitative study show that most practitioners found screening STOP patients for alcohol use meaningful and useful. The results also help with understanding why no significant differences were observed in practitioners’ likelihood of offering an alcohol intervention to eligible patients, between the practitioners at clinics randomized to the intervention or control arm. It is unlikely that practitioners will offer a brief alcohol intervention and provide an educational resource to their patients, regardless of the presence of a CDSS, if they do not perceive it as needed for their patient population. This finding contributes to the growing literature of partial implementation of technology in health service settings [57].

For this study we used the Hexagon Tool as a framework to understand the barriers and facilitators of implementing an alcohol intervention into the STOP program [45]. Even though this framework is primarily used as a planning tool to guide the selection of a program to implement, it also proved useful as a framework to understand what enabled and hindered practitioners in implementing COMBAT as intended. For interventions that have already been implemented, the Hexagon Tool helps with understanding whether or not the intervention was appropriate for the local context [45]. It is able to break down the pragmatic dilemmas—organizational (need, fit and capacity) and program specific (evidence, support and usability)—that practitioners faced, and how it affected their likelihood to deliver the intervention [45]. As a result, the Hexagon Tool is able to help us identify the gaps in the intervention and how we can improve its design for future programs.

### Limitations

There are a few limitations to our study. Interviews were conducted before we knew the trial’s results, specifically that CDSS did not increase practitioner likelihood of offering an alcohol intervention but did increase the acceptance rate of an educational alcohol resource by patients. As a result, we did not probe to understand practitioners’ perceptions on how the prompted CDSS influenced their decision to deliver the brief intervention. Moreover, lack of training was reported as a major barrier however many of the practitioner in the sample reported not having attended the training webinars that were offered. These interviews did not explore barriers to participating in these training webinars, however this is an important component to consider in future qualitative studies. The interviews also sampled the views of a small number of STOP practitioners who implemented the COMBAT initiative and there were varying degrees of fidelity to the intervention procedures. Moreover, the practitioners in our sample were primarily female. For these reasons, it is possible that the perspectives of practitioners who took part in the qualitative interviews may not reflect the views of practitioners who did not participate. However, the intensity sampling methodology we used allowed us to interview practitioners from diverse clinic settings (FHTs, CHCs, and NPLCs), study arm (intervention and control) and belonging to clinics which provided the resource frequently versus infrequently. In addition, our high response rate (92%), and the similarity of the identified themes to previous qualitative research in this field strengthens the validity of this study and supports the applicability of the results.

## Conclusions

The results of the qualitative interviews with STOP practitioners add to our understanding of factors that are necessary to successfully embed a brief alcohol intervention into an existing smoking cessation program delivered in primary care settings. Our findings suggest that the goal of an effectively embedded preventative care pathway for alcohol remains tenuous. Although more trainings and supports for health care practitioners might help integrate the delivery of brief interventions over time, the results of this study show the need to change social norms regarding alcohol in order to effectively embed alcohol interventions into a smoking cessation program in primary care clinics. It was clear that the beliefs of practitioners are reflective of a larger sociocultural context. Since 2015, Ontario has been changing its laws to make alcohol more accessible [58]. The Liquor Licence Act of Ontario (2015) was changed to allow grocery stores in Ontario to sell wine, beer and cider [58]. The 2018 campaign promised to decrease the price of beer and revamp the sales policy of alcohol to extend the hours during which alcohol can be sold in the province [58]. The 2019 Ontario budget expanded access to alcohol by allowing: drinking alcohol in parks, tailgating at sporting events, earlier opening hours for bars, and relaxing alcohol-advertising rules [58]. In this context, it seems unlikely that societal perceptions regarding alcohol in Ontario will change in a way that would encourage more health practitioners to offer patients a brief alcohol intervention. However, as previous research has found, these same policies may increase the need for such interventions [59, 60].

## Supplementary Information

**Additional file 1. **Qualitative Interview Guide. This file contains the interview guide that was used to conduct the qualitative interviews with STOP practitioners who were part of the COMBAT trial.

## Data Availability

The datasets generated and/or analysed during the current study are not publicly available but are available from the corresponding author on reasonable request.

## Abbreviations

AUDIT: Alcohol Use Disorders Identification Test
CAMH: Centre for Addiction and Mental Health
CCS: Canadian Cancer Society
CDSS: Clinical Decision Support System
CHC: Community Health Centre
COMBAT: **Comb**ined **A**lcohol and **T**obacco
FHT: Family Health Team
NPLCs: Nurse Practitioner-led Clinic
SBIRT: Screening, Brief Intervention, and Referral to Treatment
STOP: Smoking Treatment for Ontario Patients
