# Supplementary material for: Integrating a brief alcohol intervention with tobacco addiction treatment in primary care: qualitative study of health care practitioner perceptions
Source: Addict Sci Clin Pract. 2021 Mar 16;16:17. doi: 10.1186/s13722-021-00225-x (PMC7968293; doi:10.1186/s13722-021-00225-x)
Supplement: Supplementary file 1 — Additional file 1. Qualitative Interview Guide. This file contains the interview guide that was used to conduct the qualitative interviews with STOP practitioners who were part of the COMBAT trial. [file 13722_2021_225_MOESM1_ESM.pdf]

Qualitative Interview Guide

Ice Breaker

1. Can you tell me about your role at your organization and how long you have been involved in STOP?

Research Questions

2. What training, if any, have you received for addressing risky alcohol use?
    a. Probe for different levels of alcohol use (exceeding CCS guidelines, at risk of dependence, etc.)
    b. Probe for whether training impacted the way they address smoking cessation

3. Can you tell me about your experiences using the STOP portal since alcohol-related changes such as alcohol questions, educational resources, [Intervention Group only: brief intervention messaging]) were added?
    a. Gauge for positive vs. negative overall impression, comfort level

4. Can you describe your experience identifying patients (i.e. via screening questions) who drink above CCS alcohol guidelines?
    a. Probe for ease/difficulty level

5. How are patients responding to the new alcohol-related <u>questions</u> to STOP at your clinic? How relevant do you feel these questions are for your patients?

6. Intervention Group only: What are your overall impressions of the brief intervention script to help clients reduce their drinking?
    a. Probe for: clients who drink at different levels, with comorbidities, income; did it seem effective; what would make it better

7. How are patients responding to the new alcohol-related educational resources available as part of the STOP questionnaire? Probe for clients who **drink** at different levels, different socioeconomic statuses, genders.
    a. How relevant do you feel these resources are for your patients? If not relevant, probe for why, including why resources not offered
    b. How are the resources received by patients? If not received well, probe for why, including why resources not accepted

8. What are your overall impressions of the additional educational resources (i.e. sleep, stress, etc.) provided through the STOP portal?
    a. How effective, if at all, do they seem for your clients? How could they be improved?

9. How did the STOP portal function after the alcohol-related changes were added? (e.g. survey question logic, sharing of resources, [Intervention Group only: CDSS prompting])

10. How has this adaptation impacted your current workflow in the STOP program? In your practice in general?
    a. Probe: Has adaptation affected length of time to complete baseline? Has adaptation impacted how many new patients are seen? Has adaptation impacted how many patients return for follow-up visits?
    b. Probe: How does this adaptation fit with other initiatives happening in your clinic?
    c. Probe: How, if at all, has this adaptation been used for your patients not participating in the STOP program? (i.e. using same screening measures, sharing resources with non-STOP patients, etc.)

11. How prepared do you feel your clinic was to adopt the new alcohol-related changes, such as alcohol questions, educational resources, [Intervention Group only: brief intervention messaging] to STOP?
    a. Probe: sufficient staff to deliver the program; staff knowledge/training/skills in alcohol reduction, equipped to improve implementation over time)

12. What challenges and barriers to delivering the alcohol intervention as part of the ***STOP program*** did you encounter? How did you address these challenges or barriers? Please explain.

    a. Probe, if not already covered:
        i. Organizational: lack of time, resources, support, funding; other priorities; technical difficulties
        ii. Practitioner-level: lack of staff confidence, knowledge, training, difficult scoring alcohol questions
        iii. Patient-level: complex patient cases, mental health issues, other addictions, patients not ready to quit smoking and reduce alcohol consumption, patient not interested in receiving the resources
        iv. Program specific challenges: question was annoying, resources were hard to find, (Group A: screeners difficult to manually score), questionnaire changes were burdensome, etc.

13. How might we be able to improve this adaptation for you? What changes could be made to help streamline your workflow?

14. How prepared is your organization to carry out future adaptations to the STOP program?
    a. Probe: Addressing additional behaviors simultaneously with smoking cessation (i.e. first alcohol, then mood/stress/etc.)

15. How might we be able to improve this adaptation for clients?

16. Thus far, what changes have you observed in smoking cessation rates at your clinic as a result of delivering the alcohol intervention as part of ***STOP program***?

17. Thus far, what changes have you observed in alcohol consumption levels at your clinic as a result of delivering the alcohol intervention as part of ***STOP program***?

18. Thus far, what changes have you observed, if any, in client satisfaction at your clinic as a result of delivering the alcohol intervention as part of ***STOP program***?

Closing Question

19. Is there any other feedback or advice that you would like to provide for us regarding this initiative and the STOP portal adaptation?
